# MiR-155 Regulates PAD4-Dependent Formation of Neutrophil Extracellular Traps

**DOI:** 10.3389/fimmu.2019.02462

**Published:** 2019-11-01

**Authors:** Avin Hawez, Amr Al-Haidari, Raed Madhi, Milladur Rahman, Henrik Thorlacius

**Affiliations:** Section for Surgery, Department of Clinical Sciences, Malmö, Lund University, Malmö, Sweden

**Keywords:** microRNA, histone, inflammation, neutrophils, NETosis

## Abstract

Accumulating data suggest that neutrophil extracellular traps (NETs) exert a key function in several diseases. Peptidylarginine deiminase 4 (PAD4) regulates NET formation via citrullination of histones. The aim of this study was to examine the role of miR-155 in controlling PAD4-dependent generation of NETs. Bone marrow neutrophils were stimulated with PMA and MIP-2. Pre-incubation of neutrophils with translational inhibitors (cycloheximide or puromycin) markedly decreased NET formation induced by PMA or MIP-2. Neutrophil transfection with a mimic miR-155 increased PMA-induced PAD4 mRNA expression and NET formation. In contrast, transfection with an antagomiR-155 decreased induction of PAD4 mRNA and NETs in response to PMA challenge. Bioinformatical examination of PAD4 revealed a potential binding site in AU-rich elements at the 3′-UTR region. MiR-155 binding to PAD4 was examined by use of target site blockers and RNA immunoprecipitation, revealing that miR-155 regulation of PAD4 mRNA is mediated via AU-rich elements in the 3′-UTR region. In conclusion, our findings demonstrate that miR-155 positively regulates neutrophil expression of PAD4 and expulsion of extracellular traps. Thus, our novel results indicate that targeting miR-155 might be useful to inhibit exaggerated NET generation in inflammatory diseases.

## Introduction

Neutrophils are critical in the host defense against bacterial infections (1) but these cells also play a pathological role in a wide range of diseases, including sepsis, acute pancreatitis, inflammatory bowel diseases and ischemia-reperfusion injuries (2–5). Activated neutrophils undergo numerous important changes for the host defense against invading pathogens. The primary responses of neutrophils exposed to bacteria include the production of reactive oxygen species and engulfment of microbes (6). Moreover, recent findings have demonstrated that activated neutrophils also expel nuclear DNA to form web-like structures containing nuclear, cytoplasmatic and granular proteins, which are referred as neutrophil extracellular traps (NETs) (7). NETs have been documented to exert antimicrobial effects by trapping and killing bacterial and fungal invaders (7, 8). Moreover, exaggerated NET formation has been shown to cause tissue injury in models of inflammation and infection (9). Convincing data have documented that peptidylarginine deiminase (PAD) enzymes mediate citrullination, i.e., conversion of arginine to citrulline in certain proteins, including histones (10, 11). Hypercitrullination of target histone 2A, 3, and 4 causes chromatin decondensation and constitutes a key process in NET formation (12). There are five known PAD enzymes which share 70–95% amino acid identity and the calcium binding sites show very high levels of conservation with the exception of PAD6 (13). Among the five PAD enzymes expressed in humans and mice, PAD4 is highly expressed in neutrophils (14). PAD4 has been shown to be a major regulator of NET formation via deimination of histone H3 to citrullinated histone H3 causing chromatin decondensation and release (15, 16) although more efforts are needed to understand signaling pathways regulating PAD4 activity and expulsion of DNA from neutrophils.

MicroRNAs (miRs) are evolutionarily conserved, short (21–22 nucleotides), non-coding RNA molecules that post-transcriptionally regulate gene expression through complementary base-pairing to the 3′ UTR of target mRNAs (17). In general, this causes target mRNA degradation or translational inhibition, allowing miRs to regulate large parts of the cell proteome (18). Nonetheless, under certain conditions such as cellular stress or serum starvation, which puts cells in a state of quiescence, certain miRs has been shown to promote mRNA expression (19–24). The literature contains ample data indicating dramatic and global changes in miR expression in systemic inflammation and sepsis (25). Most of the work on the immune system have focused on the regulatory role of miRs on macrophages and lymphocytes and there is very limited information available regarding how miRs regulate neutrophil functions. Current data suggest that miR-223, miR-142, miR-451 can regulate neutrophil development and function (26). and one recent study reported that miR-146a can regulate the formation of NETs (27). It is well-known that miR-155 exerts multiple functions in the immune system (28). Interestingly, a previous investigation showed that miR-155 negatively controls neutrophil migration toward chemotactic stimuli (29) although the role of miR-155 in NET generation is not known.

Based on the considerations above, we hypothesized herein that miR-155 might regulate PAD4 function and expulsion of DNA from neutrophils.

## Materials and Methods

### Neutrophil Isolation

All experiments were approved by the Regional Ethical Committee for Animal Experimentation at Lund University, Sweden (permit number: M136-14). Briefly, male C57BL/6 mice (20–25 g, 8–9 weeks old) were euthanized by careful dislocation of the epiphyses (cervical dislocation). Femurs and tibia were collected in sterile Petri dishes containing ice-cold Roswell Park Memorial Institute medium 1640 (RPMI 1640, Invitrogen, Stockholm, Sweden). Bone marrow cells were flushed by using 25-gauge needle syringe filled with 2 ml of RPMI 1640 supplemented with 10% fetal bovine serum (FBS, Invitrogen) and 2 mM EDTA (Sigma-Aldrich, St. Louis, MO, US) into a 15 ml tube. After hypotonic lysis (5 ml ice-cold 0.2% NaCl, added for 45 s followed by addition of 5 ml 1.6% NaCl) cells were isolated using a Ficoll-Paque™ plus (GE Healthcare, Uppsala, Sweden). Neutrophils were separated from mononuclear cells by centrifuging at 1,600 rpm for 30 min. Pellets containing neutrophils were collected and washed with RPMI 1640. Neutrophil purity (72–74%, Supplementary Figure 1B) was confirmed by CytoFLEX flow cytometer (Becton Dickinson) using phycoerythrin (PE) conjugated anti-Ly6G (clone 1A8, BD Pharmingen) antibody. Cells were resuspended at 5 × 10^6^ cells/ml for subsequent use.

### Neutrophil Viability Assay

Cell viability of neutrophil assessed by using trypan blue dye exclusion assay. Neutrophil (5 × 10^6^ cells) were seeded in 6 well plates and allowed to adapt for 3 h and then transfected as described below. Cells were collected after 24 h by low speed centrifugation and trypan blue viability assay was performed by adding 1:1 volume samples and trypan blue dye and percentage of viable cells were estimated by hemocytometer (Supplementary Figure 1A).

### Neutrophil Transfection

Isolated neutrophils (5 × 10^6^ cells) were seeded in 6-well plates in DMEM in a total volume of 2 ml supplemented with 10% FBS and 20 ng/ml recombinant mouse G-CSF (Life Technologies, Carlsbad, CA, USA). Cells were allowed to adapt for 3 h and then transfected with mmu-miR-155-5p Mimic (50 nM), Ctrl-Mimic, antagomiR-155-5p (50 nM) or ctrl-antagomiR-155 (50 nM) using Mirus transfection reagent (Madison, WI, USA) in Opti-MEM (Life Technologies, Carlsbad, CA, USA) for 24 h. mmu-miR-155-5p Mimic, Ctrl-Mimic, antagomiR- and ctrl-antagomiR-155 were purchased from Life technologies. Cells were washed and harvested for subsequent assays. Transfection efficiency was evaluated by quantitative real-time polymerase chain reaction (qRT-PCR).

### Assessment of Cytotoxicity

Lactate dehydrogenase (LDH) leakage into the culture media due to the membrane damage was used as an indicator of drug cytotoxicity. Neutrophils were pre-incubated with 1, 10, 200, and 500 μg/ml of cycloheximide or 1 and 10 μg/ml puromycin for 30 min and then incubated for 3 h (same as stimulation time) at 37°C. The activity of LDH in the medium was determined by using LDH-Cytotoxicity Colorimetric Assay Kit II (BioVision, USA) following the manufacture's instructions. Samples without any drug were used as a low control and samples from lysed cells were used as a high control. Percentage of toxicity was calculated using the following formula: (Test sample-Low control)/(High control-Low control) X100.

### Experimental Protocol

Neutrophils non-transfected and transfected with miR-155-5p, Ctrl-miR-155-5p, antagomiR-155-5p or ctrl-antagomiR-155 were stimulated with 500 nM phorbol myristate acetate (PMA, Sigma-Aldrich, St. Louis, MO, US) for 3 h at 37°C. In certain experiments, neutrophils were pre-incubated with vehicle, 1 and 10 μg/ml of cycloheximide (Sigma-Aldrich) or puromycin (Sigma-Aldrich) for 5–30 min and then stimulated with 500 nM PMA or 100 ng/ml of MIP-2 (PeproTech, Rocky Hill, USA) for 3 h at 37°C.

### DNA-Histone Complexes

Neutrophils were stimulated with PMA in 6-well plates. NETs and residual neutrophils were then harvested by extensive pipetting and NETs were isolated by gradient centrifugation. Supernatants were discarded and pellets containing NETs were collected and resuspended in 300 ml PBS. Levels of DNA-histone complexes were quantified by use of sandwich ELISA based on monoclonal antibodies directed against histones and DNA according to the manufacturer's instructions (Cell Death Detection Elisa plus; Roche Diagnosis, Mannheim, Germany).

### Flow Cytometry

A total of 1 × 10^6^ neutrophils were stimulated with PMA or MIP-2 in suspension. Following sample centrifugation (400 g, 5 min) pellets containing neutrophils were fixed (2% formaldehyde). Cells were washed two times in 2% FBS and incubated with primary antibodies: phycoerythrin (PE) conjugated anti-Ly6G (clone 1A8, BD Pharmingen), fluorescein isothiocyanate (FITC) conjugated anti-MPO antibody (mouse: ab90812 Abcam, Cambridge, MA) and rabbit anti-citH3 (citrulline 2, 8, 17, ab5103; Abcam, Cambridge, MA) in 5% donkey serum. After washing two times, cells were incubated with rat anti-rabbit allophycocyanin (APC) conjugated secondary antibody (A-21038, Thermo Fisher Scientific, Rockford, USA). Neutrophils were analyzed using standard settings on a CytoFLEX flow cytometer (Becton Dickinson, Mountain View, CA, USA). Neutrophils were defined as Ly6G+ cells. MPO and citrullinated H3 determined on gated Ly6G positive cells.

### Confocal Microscopy

Neutrophils (0.5 × 10^6^) were seeded over coverslips in a 24-well plate containing 10% FBS and 20 ng/ml recombinant mouse G-CSF (Life technologies) and then stimulated with PMA or MIP-2 as described above. After stimulation, neutrophils were washed and fixed with 4% paraformaldehyde for 10 min and then permeabilized with 0.1% Triton X-100, then washed and blocked with 1% BSA for 45 min followed by incubation with specific primary antibodies: FITC-conjugated anti-mouse MPO antibody (mouse: ab90812 Abcam, Cambridge, MA) and rabbit anti-mouse citrullinated histone H3 antibody (citrulline 2, 8, 17, ab5103; Abcam, Cambridge, MA) for 2 h. Coverslips were washed and incubated with rat anti-rabbit secondary antibody (A-21038, Thermo Fisher Scientific, Rockford, USA) for 1 h and the nucleus were counter-stained with Hoechst 33342 (1 μg/ml, Thermo Fisher Scientific) for 10 min. Samples were then mounted using Prolong™ Diamond Antifade Mountant (Thermo Fisher Scientific). Confocal microscopy images were taken by a ×63 oil immersion objective (numeric aperture = 1.25) in LSM 800 confocal (Carl Zeiss, Jena, Germany. The pinhole was set ~ 1 airy unit and the scanning frame was 1,024 × 1,024 pixels. ZEN2012 software were used to processed images. For quantification, H3cit covered area was considered as NETs area. In brief, H3cit channel was split from 16-bit image and lower threshold level was set to 12 and then percentage of H3cit covered area was quantified in four random fields using Fiji software.

### qRT-PCR

Total RNA was isolated from neutrophils using Direct-Zol RNA MiniPrep (Zymo Research, Irvine, CA, USA) kit following the manufacturer's instructions. Total RNA concentration was determined using Nanodrop spectrophotometer at 260 nm absorbance. cDNA was synthesized by Mir-X miRNA First-Strand synthesis kit (Clontech, CA, USA) in a final reaction volume of 10 μL according to the manufacturer's instructions and qRT-PCR was conducted in a final volume of 25 μL using SYBR Green dye (Takara Bio, USA) for relative expression of PAD4 and miR-155-5p. The PCR primers used were as follows; mmu-miR-155-5p sense; 5′-UUAAUGCUAAUUGUGAUAGGGGU-3′, PAD4 sense; 5′-TGTGACCCGAAAGCTCTA-3′, PAD4 antisense; 5′-CTGCTGGAGTAACCGCTATT-3′, U6 sense; 5′-GCTTCGGCAGCACATATACTA-3′, U6 antisense; 5′-CGAATTTGCGTGTCATCCTTG-3′, GAPDH sense; 5′-GTCCCAGCTTAGGTTCATAG-3′, GAPDH antisense; 5′-GATGGCAACAATCTCCACTTTG-3′. Expression of miR-155-5p relative to house-keeping gene U6 and expression of PAD4 mRNA relative to house-keeping gene GADPH were determined using 2^−ΔΔ*CT*^ method.

### Western Blot

Protein concentration in neutrophils was measured by Pierce bicinchoninic acid (BCA) protein assay kit (Thermo Fisher Scientific). 20 μg of total protein was added in each lane and separated on 8–16% Mini-PROTEAN® TGX Stain-Free™ Gels (Bio-Rad). Proteins were then transferred to polyvinylidene fluoride membranes (Novex, San Diego, CA, USA). Prior to blotting, total protein gel image was obtained by use of Bio-Rad's stain-free gel chemistry. Next, TBS/Tween-20 buffer (5% non-fat milk powder) was used to block non-specific bindings on the membranes. Protein immunoblots were performed using rabbit monoclonal citrullinated anti-Histone H3 (1:2,000, ab5103, Abcam) or anti-PAD4 (1:1,000, ab214810, Abcam) and incubated overnight at 4°C. Membranes were incubated with goat anti-rabbit secondary antibody (1:2,000) conjugated with horseradish peroxidase at room temperature for 1 h. Protein bands were normalized to stain-free total protein loads of respective lanes (Supplementary Figure 4). Bands images were visualized by use of the Bio-Rad ChemiDoc™ MP imaging system and examined by Image Lab™ software version 5.2.1.

### Target Site Blockers (TSBs)

MiRs usually regulate multiple targets, therefore TSB are used to validate their binding sites. To predict the binding sites for miR-155-5p at the 3′-UTR of PAD4 mRNA, we used the RNAhybrid web-based bioinformatics target prediction algorithm (http://bibiserv.techfak.uni-bielefeld.de/rnahybrid). RNAhybrid predicted four binding sites (Supplementary Figure 3), however, a strong line of evidence suggests that miR-155-5p play a crucial role in the regulation of vital proteins by binding to ARE sites in mRNAs specifically AUUA and AUUUA motifs and studies were thus limited ARE sites. Only one binding site was identified based on complementary-base pair bioinformatics analysis. To examine the role of this binding site, a TSB (22 nucleotides) was designed to bind to sequences overlapping with the miR-155-5p ARE sites in the 3′-UTR of PAD4 mRNA. In order to enhance target affinity and selectivity the blocker was synthesized as fully phosphorothiolated Locked Nucleic Acids (LNA) in the DNA sequences. The target site blockers TSB_PAD4_miR-155-5p; 5′-TTAATTTTTATTAAATATATAT-3′ and TSB negative control _PAD4_miR-155-5p; 5′-TAACACGTCTATACGCCCA-3′ were co-transfected with the miR-155-5p mimic in different concentrations (12.5–50 nM) in neutrophils. RT-qPCR was used to measure levels of PAD4 mRNA and predicted target was functionally validated by use of RNA immunoprecipitation (RIP) assays.

### RIP Assay

For experimental validation of miR-155-5p binding to PAD4 mRNA, RIP assays were performed to immune-precipitate Ago protein complex that contains functionally related miRNAs:mRNAs complexes using EZ-Magna RIP kit (Millipore, Billerica, MA, USA) as previously described (19). RNA was then extracted using Direct-zol RNA extraction kit and 0.5 μg total RNA was used for cDNA synthesis. RT- qPCR was used ro measure relative expression of miR-155-5p and PAD4 mRNA in Ago2 immunoprecipitates.

### Statistics

Data are presented as mean values ± standard error of the mean (SEM). For statistical analysis Kruskal-Wallis one-way ANOVA on ranks, followed by multiple comparisons (Dunnett's methods) was used. *P*-value less than 0.05 was considered statistically significant and *n* represents the number of experiments in each group.

## Results

### Net Formation Is Dependent on Protein Translation

**Figure 1:**
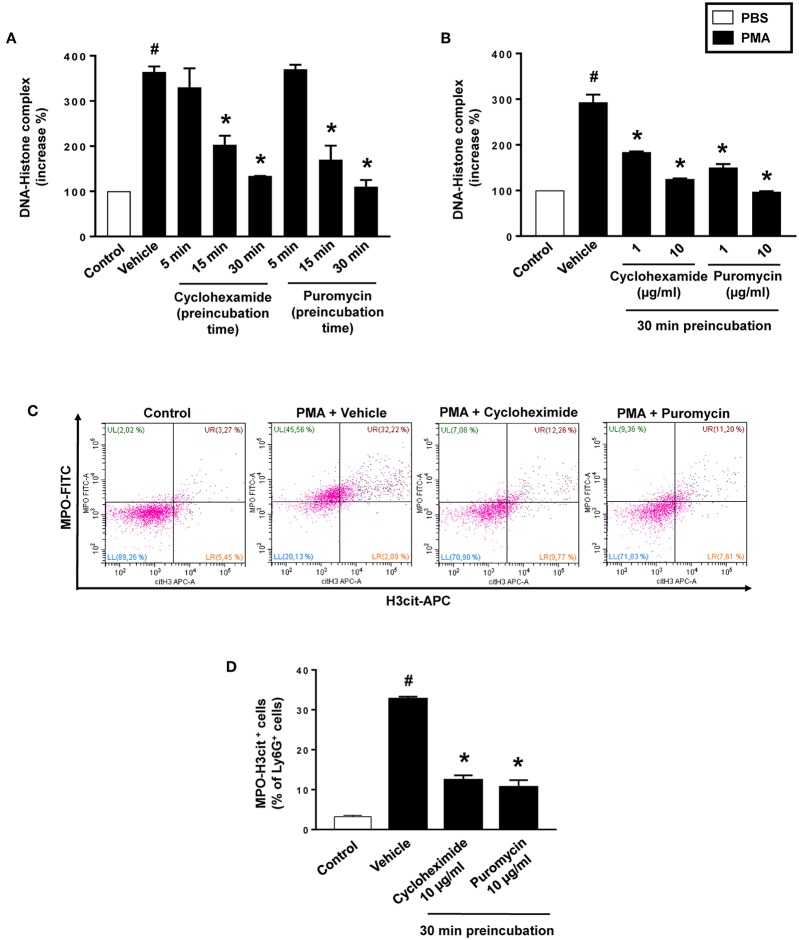
NET formation in neutrophils. **(A)** Neutrophils were preincubated with 10 μg/ml of cycloheximide or puromycin for 5–30 min and stimulated with PMA (500 nM) for 3 h at 37°C. **(B)** Neutrophils were preincubated with indicated concentrations of cycloheximide or puromycin for 30 min and stimulated with PMA (500 nM). DNA-histone complexes were quantified in supernatants by ELISA. **(C)** Levels of citrullinated Histone H3 and MPO in neutrophils (Ly6G+ cells) were determined by FACS. Neutrophils were preincubated with 10 μg/ml of cycloheximide or puromycin for 30 min and stimulated with PMA (500 nM). **(D)** Aggregate data of flow cytometry. Data represent mean ± SEM and *n* = 5. ^#^*P* < 0.05 vs. control and *P < 0.05 vs. vehicle.

**Figure 2:**
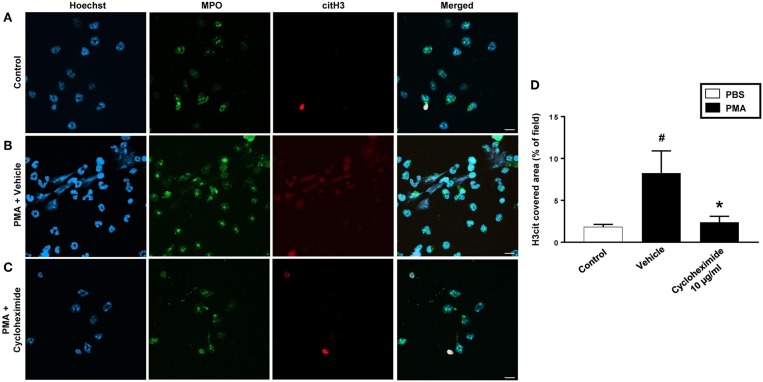
NETs were visualized by confocal immunofluorescence microscopy in isolated neutrophils. Cells were preincubated with or without 10 μg/ml cycloheximide and stimulated with PMA (500 nM) for 3 h at 37°C. **(A–C)** Neutrophils were immune-stained with antibodies to myeloperoxidase (MPO-green), citrullinated Histone H3 (citH3-red), hoechst-blue to counterstain DNA. Non-stimulated cells served as a control. Images are representative of four independent experiments. Scale bars = 10 μm. **(D)** NETs covered area per field of view were quantified using Fiji and expressed as percentage of H3cit covered area. Data represent mean ± SEM and *n* = 4. ^#^*P* < 0.05 vs. control and *P < 0.05 vs. Vehicle.

**Figure 3:**
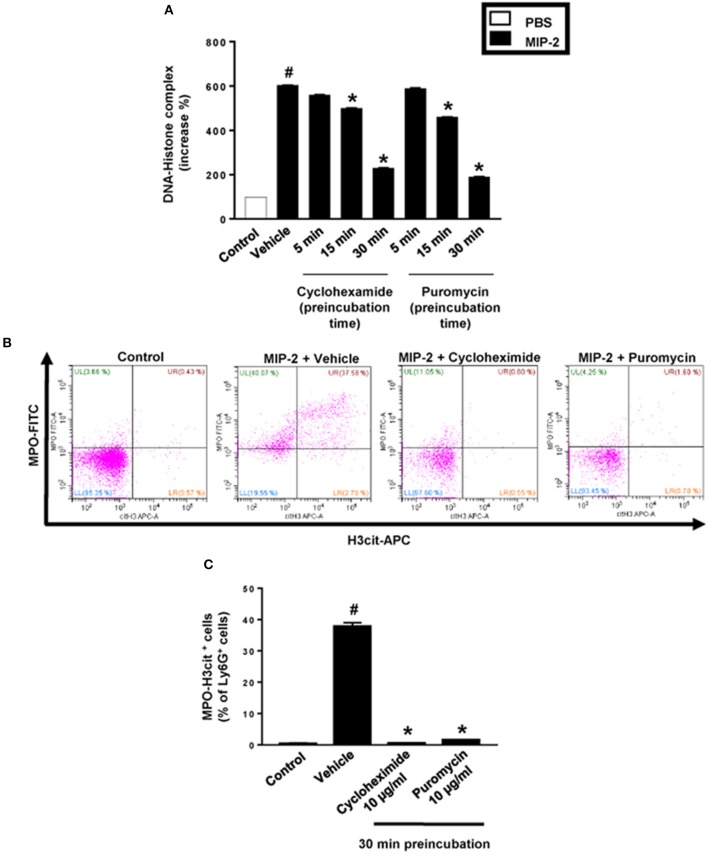
NET formation in neutrophils. **(A)** Neutrophils were preincubated with 10 μg/ml of cycloheximide or puromycin for 5–30 min and stimulated with MIP-2 (100 ng/ml) for 3 h at 37°C. DNA-histone complexes were quantified in supernatants by ELISA. **(B)** Levels of citrullinated Histone H3 and MPO in neutrophils (Ly6G+ cells) were determined by FACS. Neutrophils were preincubated with 10 μg/ml of cycloheximide or puromycin for 30 min and stimulated with MIP-2 (100 ng/ml). **(C)** Aggregate data of flow cytometry. Data represent mean ± SEM and *n* = 5. ^#^*P* < 0.05 vs. control and *P < 0.05 vs. vehicle.

**Figure 4:**
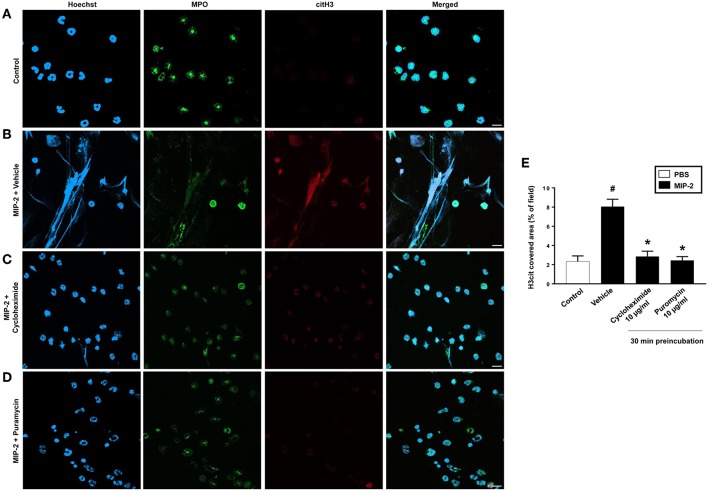
NETs were visualized by confocal immunofluorescence microscopy in isolated neutrophils. Cells were preincubated with or without 10 μg/ml cycloheximide or puromycin and stimulated with MIP-2 (100 ng/ml) for 3 h at 37°C. **(A–D)** Neutrophils were immune-stained with antibodies to myeloperoxidase (MPO-green), citrullinated Histone H3 (citH3-red), hoechst-blue to counterstain DNA. Non-stimulated cells served as a control. Images are representative of four independent experiments. Scale bars = 10 μm. **(E)** NETs covered area per field of view were quantified using Fiji and expressed as percentage of H3cit covered area. Data represent mean ± SEM and *n* = 4. ^#^*P* < 0.05 vs. control and *P < 0.05 vs. vehicle.

PMA stimulation of isolated neutrophils markedly increased DNA-histone complex formation (Figures 1A,B). Pre-incubation of neutrophils with 1 and 10 μg/ml of cycloheximide or puromycin for 30 min significantly decreased PMA-induced generation of DNA-histone complexes in neutrophils (Figure 1B). In separate experiments, it was found that 30 min, but not 5 min, of pre-incubation with cycloheximide or puromycin decreased DNA-histone complex formation in neutrophils exposed to MIP-2 (Figure 3A). Notably, preincubation of neutrophils with 10 μg/ml of cycloheximide or puromycin for only 5 min had no effect on DNA-histone complex formation after challenge with PMA (Figure 1A). Citrullinated histone H3 is an indicator of NETs formation (30). By use of flow cytometry, we quantified expression of MPO and citrullinated histone H3 on neutrophils. PMA stimulation provoked a clear-cut upregulation of MPO and citrullinated histone H3 on neutrophils (Figures 1C,D). Pre-incubation of neutrophils with 10 μg/ml of cycloheximide or puromycin for 30 min decreased PMA-induced expression of MPO and citrullinated histone H3 on neutrophils by 69 and 75%, respectively (Figures 1C,D). Moreover, 30 min of pre-incubation with cycloheximide or puromycin significantly reduced MIP-2-provoked neutrophil expression of MPO and citrullinated histone H3 (Figures 3B,C). Confocal microscopy showed that that PMA stimulation triggered the formation of fibrillar DNA and web-like structures co-localizing with MPO and citrullinated histone H3 (Figures 2A,B). Indeed, pre-incubation of neutrophils with 10 μg/ml of cycloheximide for 30 min inhibited PMA-triggered generation of these DNA structures expressing MPO and citrullinated histone H3 (Figures 2C,D). Notably, 30 min of pre-incubation with cycloheximide or puromycin abolished MIP-2-provoked generation of DNA structures containing MPO and citrullinated histone H3 (Figure 4). Neutrophil viability was not affected by the used doses of cycloheximide and puromycin (Supplementary Figure 2).

### Neutrophil Expression of miR-155 and PAD4 mRNA

**Figure 5:**
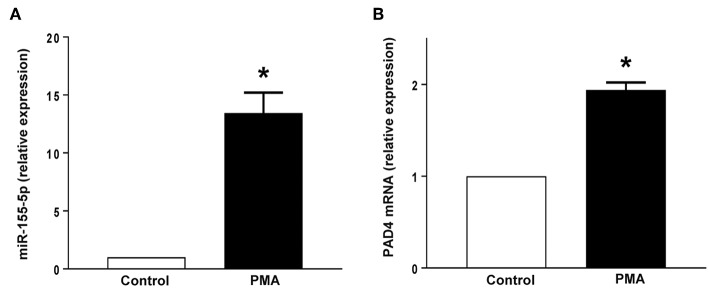
**(A)** miR-155-5p expression and **(B)** PAD4 mRNA expression. Neutrophils were stimulated with or without PMA (500 nM). Relative expression was determined by qRT-PCR. U6 and GAPDH were used as a house-keeping genes to normalize miR-155-5p and PAD4 expression, respectively. Relative expression was determined using 2^−ΔΔ*CT*^ method. Data represent mean ± SEM and *n* = 5. *P < 0.05 vs. control.

**Figure 6:**
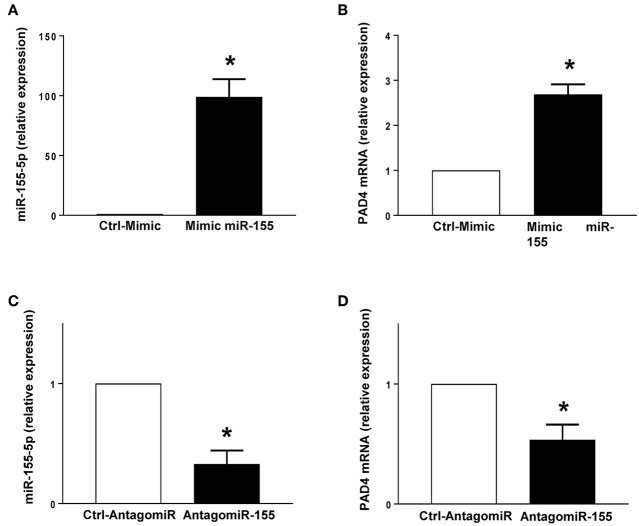
Mimic miR-155 upregulates and AntagomiR-155 downregulates PAD4 mRNA expression in neutrophils. **(A)** miR-155-5p and **(B)** PAD4 mRNA expression in cells transfected with Ctrl-Mimic and miR-155 mimic. **(C)** miR-155-5p and **(D)** PAD4 mRNA expression in cells transfected with antagomiR-Ctrl and antagomiR-155-5p. Relative expression was determined by qRT-PCR. U6 and GAPDH were used as a house-keeping genes to normalize miR-155-5p and PAD4 expression, respectively. Relative expression was determined using 2^−ΔΔ*CT*^ method. Data represent mean ± SEM and *n* = 5. *P < 0.05 vs. control.

PMA stimulation significantly increased the expression of miR-155 and PAD4 mRNA in neutrophils (Figures 5A,B). Transfection with mimic miR-155 markedly enhanced neutrophil baseline levels of miR-155 and PAD4 mRNA (Figures 6A,B). Knocking down miR-155-5p with an antagomiR decreased expression of miR-155 and PAD4 mRNA by more than 67% and 46% respectively in neutrophils (Figures 6C,D).

### PMA-Induced mRNA Expression of mIR-155 and PAD4 in Neutrophils

**Figure 7:**
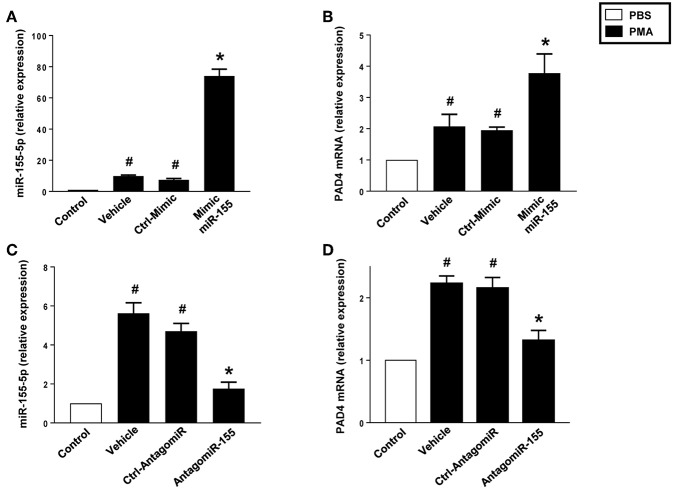
Mimic miR-155 upregulates and AntagomiR-155 downregulates PAD4 mRNA expression in neutrophils stimulated with PMA (500 nM). **(A)** miR-155-5p and **(B)** PAD4 mRNA expression in cells transfected with Ctrl Mimic and mir-155 mimic. **(C)** miR-155-5p and **(D)** PAD4 mRNA expression in cells transfected with Ctrl-antagomiR and antagomiR-155-5p. Relative expression was determined by qRT-PCR. U6 and GAPDH were used as a house-keeping genes to normalize miR-155-5p and PAD4 expression, respectively. Relative expression was determined using 2^−ΔΔ*CT*^ method. Data represent mean ± SEM and *n* = 5. ^#^*P* < 0.05 vs. control and *P < 0.05 vs. vehicle.

Challenge with PMA increased miR-155 and PAD4 in neutrophils treated with vehicle or transfected with a control mimic (Figures 7A,B). Notably, transfection of neutrophils with mimic miR-155 markedly potentiated PMA-induced levels of miR-155 (Figure 7A) and PAD4 mRNA (Figure 7B) in neutrophils. As shown in Figures 7C,D, PMA challenge significantly increased miR-155 and PAD4 mRNA levels in neutrophils treated with vehicle or transfected with a control-antagomiR. Transfection with antagomiR-155 attenuated mRNA expression of miR-155 by 79% and PAD4 by 74% in neutrophils exposed to PMA (Figures 7C,D).

### miR-155 Regulates Net Formation

**Figure 8:**
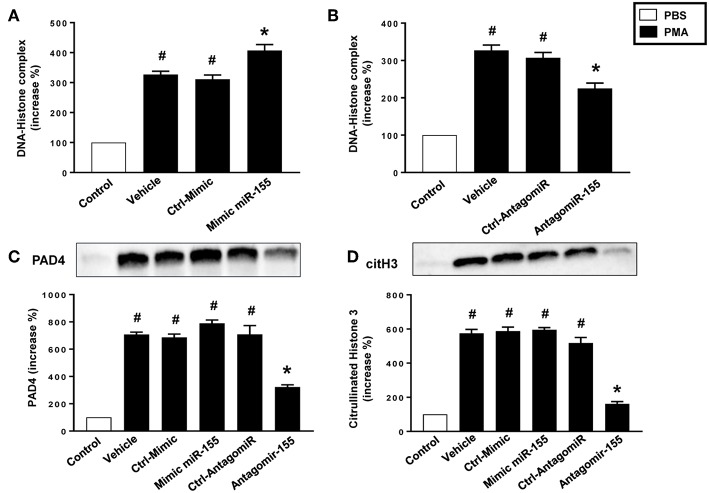
Levels of DNA histone complexes in neutrophils transfected with **(A)** Ctrl Mimic or mir-155 mimic and **(B)** Ctrl-antagomiR or antagomiR-155-5p. Expression of **(C)** PAD4 and **(D)** citH3 protein as determined by western blot and aggregate data showing PAD4 and citH3 protein expression normalized to the total protein of the respective lane. Band intensity was quantified by using Image Lab™ software. Western blots are representative of 4 independent experiments. Data are expressed as mean ± SEM and *n* = 4–5 and represented as fold change. ^#^*P* < 0.05 vs. control and *P < 0.05 vs. vehicle.

**Figure 9:**
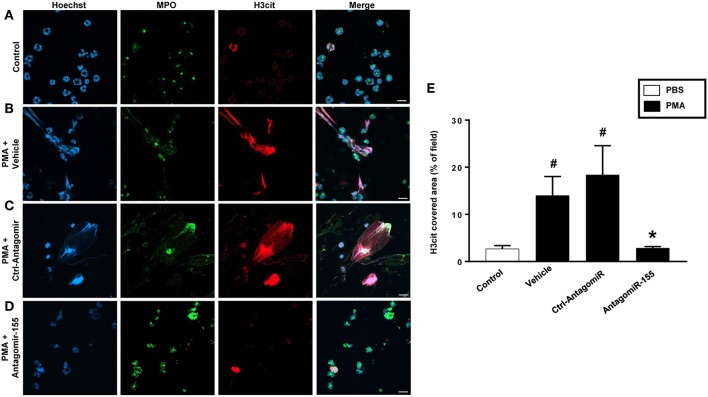
NETs were visualized by confocal immunofluorescence microscopy in neutrophils transfected with ctrl-antagomiR or antagomiR-155-5p and stimulated with PMA (500 nM). **(A–D)** Neutrophils were immune-stained with antibodies to myeloperoxidase (MPO-green), citrullinated Histone H3 (citH3-red), Hoechst-blue to counterstain DNA. Non-stimulated cells served as a control. Images are representative of four independent experiments. Scale bars = 10 μm. **(E)** NETs covered area per field of view were quantified using Fiji and expressed as percentage of H3cit covered area. Data represent mean ± SEM and *n* = 4. ^#^*P* < 0.05 vs. control and *P < 0.05 vs. Ctrl-AntagomiR.

As expected, stimulation of neutrophils with PMA markedly increased DNA-histone complex formation (Figures 8A,B). Notably, transfection of neutrophils with mimic miR-155 markedly increased PMA-induced levels of DNA-histone complexes (Figure 8A). In contrast, knocking down miR-155 in neutrophils with an antagomiR significantly reduced the formation of DNA-histone complexes triggered by PMA (Figure 8B). PMA challenge increased protein levels of PAD4 and citrullinated histone H3 in neutrophils (Figures 8C,D). Transfection of neutrophils with mimic miR-155 had no effect on PMA-induced protein levels of PAD4 and citrullinated histone H3 in neutrophils (Figures 8C,D). Transfection with antagomiR-155 significantly decreased neutrophil protein levels of PAD4 (Figure 8C) and citrullinated histone H3 (Figure 8D). Confocal fluorescence microscopy was used to image DNA, MPO and citrullinated histone H3 and their co-localization. PMA challenge caused the clear-cut formation of DNA fibrillar network co-localizing with MPO and citrullinated histone H3 (Figure 9B) in compare to control (Figure 9A), suggesting formation of NETs. Transfection with ctrl-antagomir had no effect on PMA-induced generation of NETs (Figure 9C). In contrast, it was found that transfection with antagomiR-155 greatly decreased PMA-induced generation of NETs (Figures 9D,E).

### Target Prediction of miR-155

**Figure 10:**
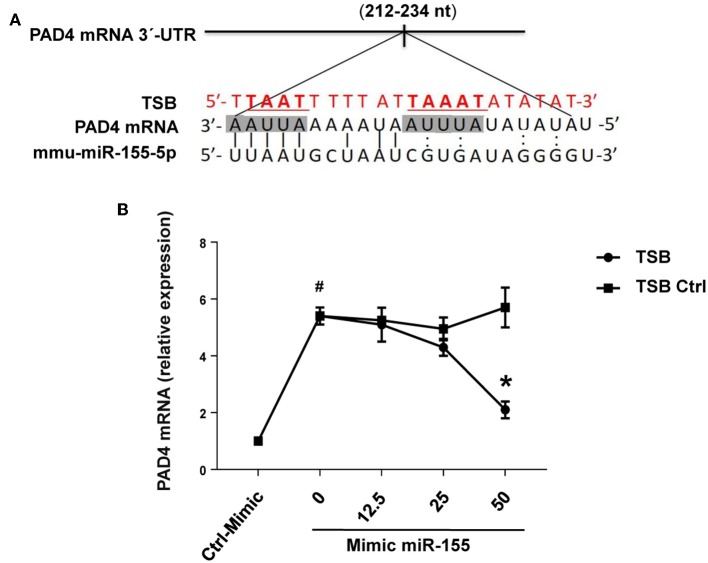
PAD4 is a direct target of miR-155-5p. **(A)** Predicted target sites of miR-155-5p in PAD4 mRNA 3-UTR sequence containing an ARE motifs (AAUUAAAAAU, AUUUA) are depicted in a shaded box. The seeding region of miR-155-5p complementary to ARE motifs was blocked using TSB, red sequence as described in section Materials and Methods. **(B)** TSB dose-dependently reversed the effect of miR-155-5p mimic on PAD4 mRNA expression in isolated neutrophils. GAPDH was used as a house-keeping genes to normalize PAD4 expression. Relative expression was determined using 2^−ΔΔ*CT*^ method. Data represent mean ± SEM and *n* = 5. ^#^*P* < 0.05 control-mimic and *P < 0.05 control TSB.

RNAhybrid target prediction analysis algorithm suggested one potential target in PAD mRNA containing miR-155 recognition sites (Figure 10A). The function of this site was studied using a target-specific blocker (TSB). As shown above, transfection with mimic miR-155 increased PAD4 mRNA expression in neutrophils (Figure 10B). Notably, co-incubation with TSB reduced expression of PAD4 mRNA in neutrophils transfected with mimic miR-155 in a dose-dependent manner (Figure 10B), suggesting that miR-155 interacts with this specific target site.

### PAD4 Is a Direct Target and Associated With mIR-155

**Figure 11:**
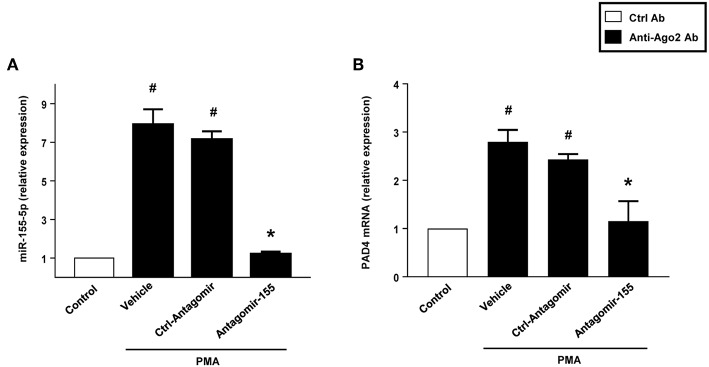
Neutrophils were transfected with ctrl-antagomiR, antagomiR-155-5p and then stimulated with or without PMA (500 nM). The amount of miR-155-5p and PAD4 mRNA were determined in input RNA by qRT-PCR in RIP assays. AntagomiR-155-5p decreased relative enrichment of **(A)** miR-155-5p and **(B)** PAD4 mRNA in Ago2 immunoprecipitates. Data are presented as fold change compared to anti-IgG ctrl. U6 and GAPDH were used as a house-keeping genes to normalize miR-155-5p and PAD4 expression, respectively. Relative expression was determined using 2^−ΔΔ*CT*^ method. Data are expressed as mean ± SEM and *n* = 5. ^#^*P* < 0.05 vs. control Ab and *P < 0.05 vs. anti-Ago2 Ab-Ctrl-antagomiR treated cells.

RIP with anti-Ago-2 beads was used to examine the potential association between miR-155 and PAD4 mRNA. Ago2 protein is the catalytic protein in RISC (31–33). It was observed that miR-155 was enriched (8-fold) in Ago2-containing miRNPs relative to control IgG immunoprecipitates (Figure 11A). In contrast, in neutrophils transfected with antagomiR-155, levels of miR-155 was reduced by 85% (Figure 11B). Compared to control IgG, the anti-Ago2 antibody pulled down 2.8-fold more PAD4 mRNA. Also, we found that knocking down miR-155 decreased PAD4 mRNA by 60% (Figure 11B).

## Discussion

NETs have emerged as an important component in both infectious and non-infectious diseases although the mechanisms regulating formation of these traps have been controversial. This study shows that protein translation contributes to NETosis. Moreover, these results suggest that miR-155 can promote induction of NETs by targeting specific sites on PAD4 mRNA. Thus, our findings suggest that miR-155 could be a useful target to antagonize NET formation in acute inflammation.

NETosis has been demonstrated to play important roles in innate immunity and is a topic of intense on-going research although the literature on the mechanisms of NET formation, such as transcription and translation, is complicated and partly contradictory. Herein, we found that translational inhibitors cycloheximide and puromycin decreases PMA-induced generation of NETs in a dose and time-dependent manner. This finding is in contrast to three previous studies reporting that there is no requirement of protein translation in the induction of NETs (34–36), which could be related to differences in methodology. We used three different approaches to quantify NETs, including DNA-histone complex formation, surface co-expression of MPO and citrullinated histone H3 as well as confocal microscopy co-localizing DNA, MPO and citrullinated histone H3 to show that protein translation contributes to NET generation. The studies reporting no role of protein translation in NET formation used DNA binding dyes, such as Sytox Green and Orange, to measure NETosis, which are fraught with significant drawbacks. For example, Sytox Green that is described as cell-impermeable by its manufacturer has been demonstrated to generate abundant non-specific false positive fluorescence signal with respect to NET formation (36). They could document a rapid and dose-dependently leakage of Sytox Green into living cells with a significant increase detected after only 15 min using just 100 nM of this dye (36), which is a 10–50 times lower concentration than those used in two of the studies mentioned above (34, 35). To further illustrate the problems with the use of these DNA binding dyes is that the study by Sollberger et al. (34) found no effect of actinomycin D on NET formation whereas Khan et al. (37) found a dose-dependent inhibitory effect of actinomycin D on NET generation leading to diametrically opposite conclusions about the role of transcription in NET generation. Moreover, the study by Sollberger et al. (34) pre-incubated neutrophils for only 5 min with cycloheximide and stimulated neutrophils for 15 h with PMA. Considering our finding that 5 min is too short time for pre-incubation with cycloheximide and that a long stimulation period (15 h) increases the risk of leakage and non-specific fluorescence signal it is perhaps not surprising that no effect of cycloheximide on NET formation was observed in that study. Nonetheless, we also confirmed that cycloheximide and puromycin also decreased NETosis in response to MIP-2 stimulation, further supporting the notion that protein translation is an integral part of NET formation. In this context, it is also interesting to note that a previous study showed that cycloheximide markedly reduces endotoxin-induced deimination of histone H3 (35), which is a critical component in NET generation. Together, these findings clearly show that NET formation is dependent on *de novo* gene expression.

Accumulating data have shown that miRs is a powerful group of short noncoding RNAs controlling numerous of cellular functions, including adhesion, apoptosis, growth, differentiation, migration and proliferation (38, 39). Accumulating data suggest that miR-155 is a major regulator of the immune system (40–42). Herein, we found that PMA not only trigged NETosis but also increased levels of PAD4 mRNA in neutrophils. Considering the important role of PAD4 in the process of NET formation (43), it was of great interest to study whether miR-155 can regulate PAD4 expression in neutrophils. In most cases, miRNAs inhibits translation of genes although emerging studies have demonstrated that miRs can also enhance gene translation in cells exposed to stress, including serum starvation (20, 24). Indeed, transfection with a mimic miR-155 and an antagomiR-155 increased and decreased PAD4 mRNA expression, respectively, in neutrophils, indicating that miR-155 is a potent regulator of PAD4 in neutrophils. On one hand, it was observed that transfection with mimic miR-155 increased not only protein levels of PAD4 but also DNA-histone complexes in neutrophils activated by PMA. On the other hand, transfection with antagomiR-155 reduced both PAD4 and DNA-histone complex levels in PMA-stimulated neutrophils. These findings indicate that miR-155 controls PAD4-dependent NET formation. This notion is also in line with our findings showing that mimic miR-155, antogomiR-155 inhibited and promoted, respectively, PMA-induced neutrophil formation of DNA structures with MPO, and citrullinated histone H3 further supporting the concept that miR-155 regulates NET generation. In addition, a recent study reported that PMA-induced generation of NET is increased in miR146a gene-deficient neutrophils, lending support to the notion that miRs are involved in the regulation of NETosis.

Convincing data have shown that 3′-UTR region of mRNA is involved in positive effects of miRs on mRNA translation (20, 23, 24). Herein, a bioinformatical study was conducted to examine if PAD4 is a potential target of miR-155. The analysis was focused on AU-rich elements (AUUA and AUUUA) in the 3′-UTR region considering that these are critical in promoting translation of mRNA (21, 44–47). The analysis revealed one region complementary to the seeding region of miR-155 with 6′-mer perfect binding. Notably, our data shows that transfection with a specific target site blocker dose-dependently reduced mimic miR-155-induced expression of PAD4 mRNA expression, indicating that this specific ARE constitute a functional miR-155 target. Thus, our results identifies a new site on PAD4 mRNA controlling translational activation by miR-155. Our present observations are in line with a previous study reporting that RhoA gene expression and migration of colon cancer cells are enhanced by miR-155 (19). In addition, the present results demonstrating that neutrophils exhibited enrichment of PAD4 mRNA and miR-155p in Ago2 protein further supports the conclusion that miR-155 interacts with PAD4 mRNA. In this context, it should be noted that miRs are capable of acting on multiple genes. In fact, enhanced gene translation can also be supported via AREs-binding molecules, Including TIA-1, TTP and Hur via indirect or direct binding of miRs (48, 49). For example, it has been reported that miR-155 increase TNFα mRNA stability and transcription via Hur in activated macrophages (50). The significance of such alternative targets of miR-155 in neutrophils requires further studies in the future.

In conclusion, our results demonstrate that miR-155 positively regulate PAD4 activity and expulsion of extracellular traps from neutrophils. In addition, we found that a specific ARE element in the 3′-UTR region of PAD4 mRNA was responsible for mediating miR-155-induced PAD4 gene expression. These findings not only elucidate the importance of translation in NET formation but might also help to develop effective strategies against NET-dependent tissue injury in acute inflammation.

## Data Availability Statement

The datasets generated for this study are available on request to the corresponding author.

## Ethics Statement

The animal study was reviewed and approved by Regional Ethical Committee for Animal Experimentation at Lund University, Sweden (permit number: 136-14).

## Author Contributions

AH, AA-H, RM, and MR performed experiments, interpreted results, and contributed to the writing. HT conceived and designed the study and contributed to the writing. All authors approved the final manuscript.

### Conflict of Interest

The authors declare that the research was conducted in the absence of any commercial or financial relationships that could be construed as a potential conflict of interest.
