# Supplementary figures and images for: MiR-155 Regulates PAD4-Dependent Formation of Neutrophil Extracellular Traps
Source: Front Immunol. 2019 Nov 1;10:2462. doi: 10.3389/fimmu.2019.02462 (PMC6838784; doi:10.3389/fimmu.2019.02462)

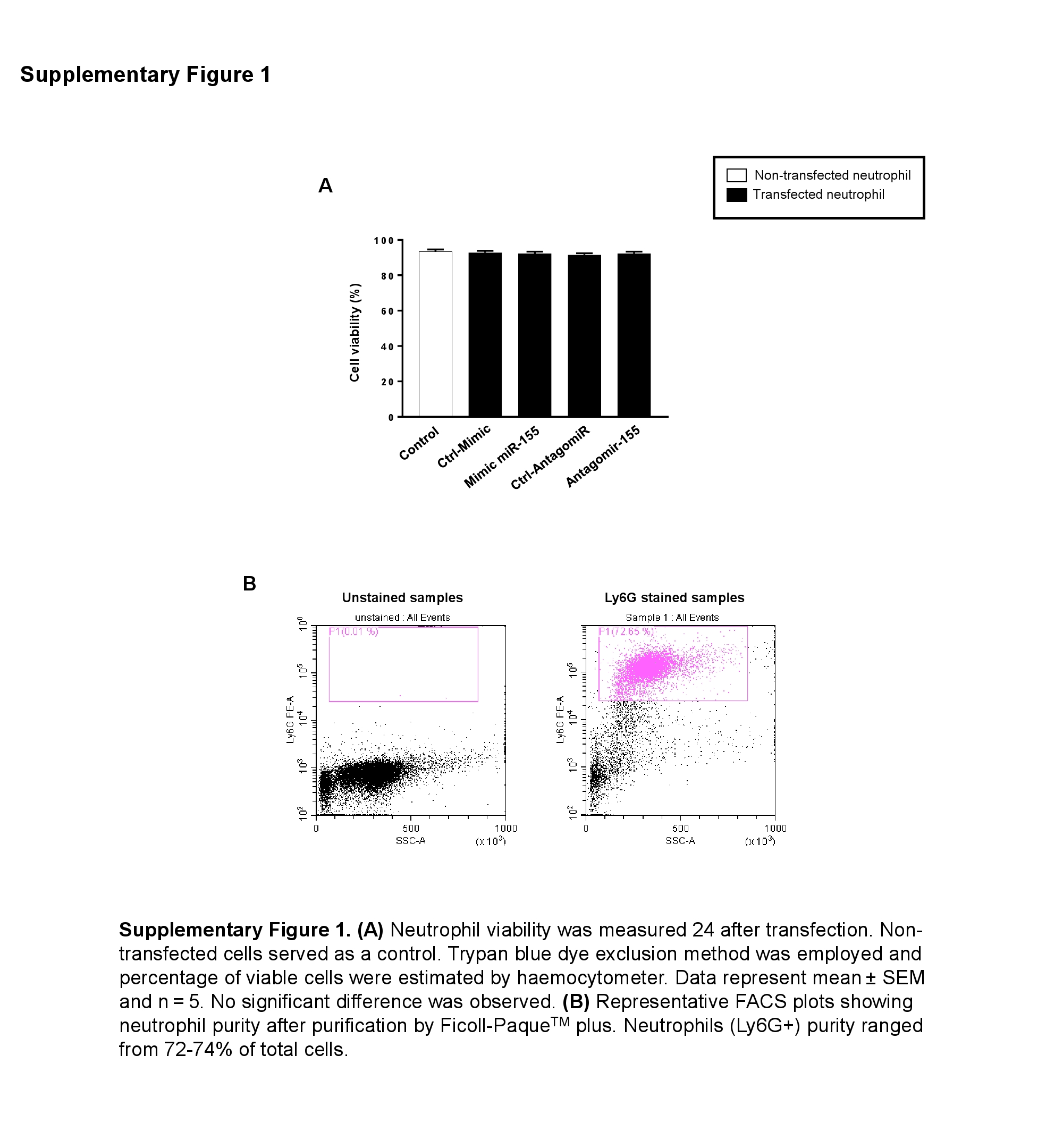

Supplement: Supplementary file 1 [file Image_1.tiff]

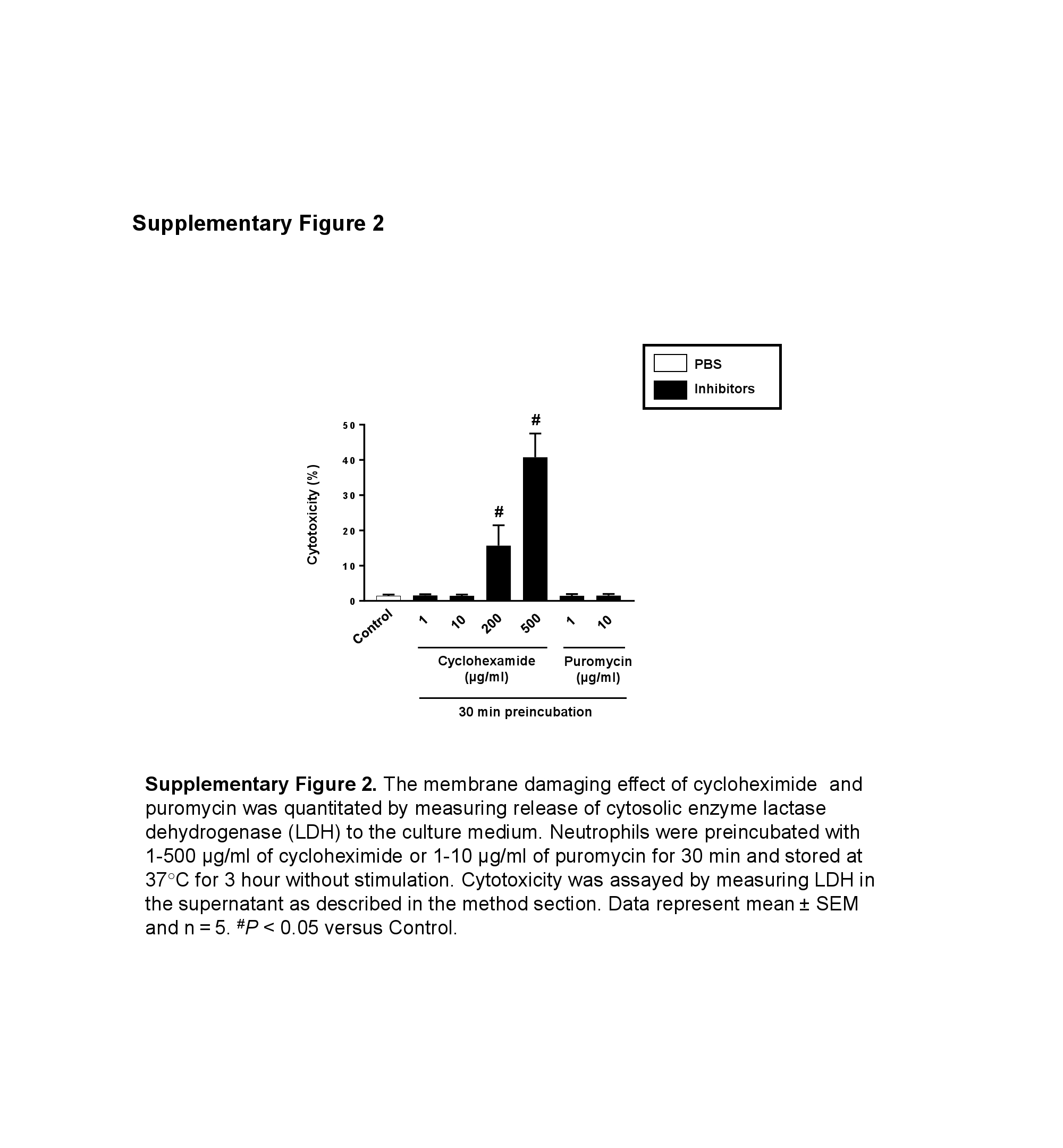

Supplement: Supplementary file 2 [file Image_2.tiff]

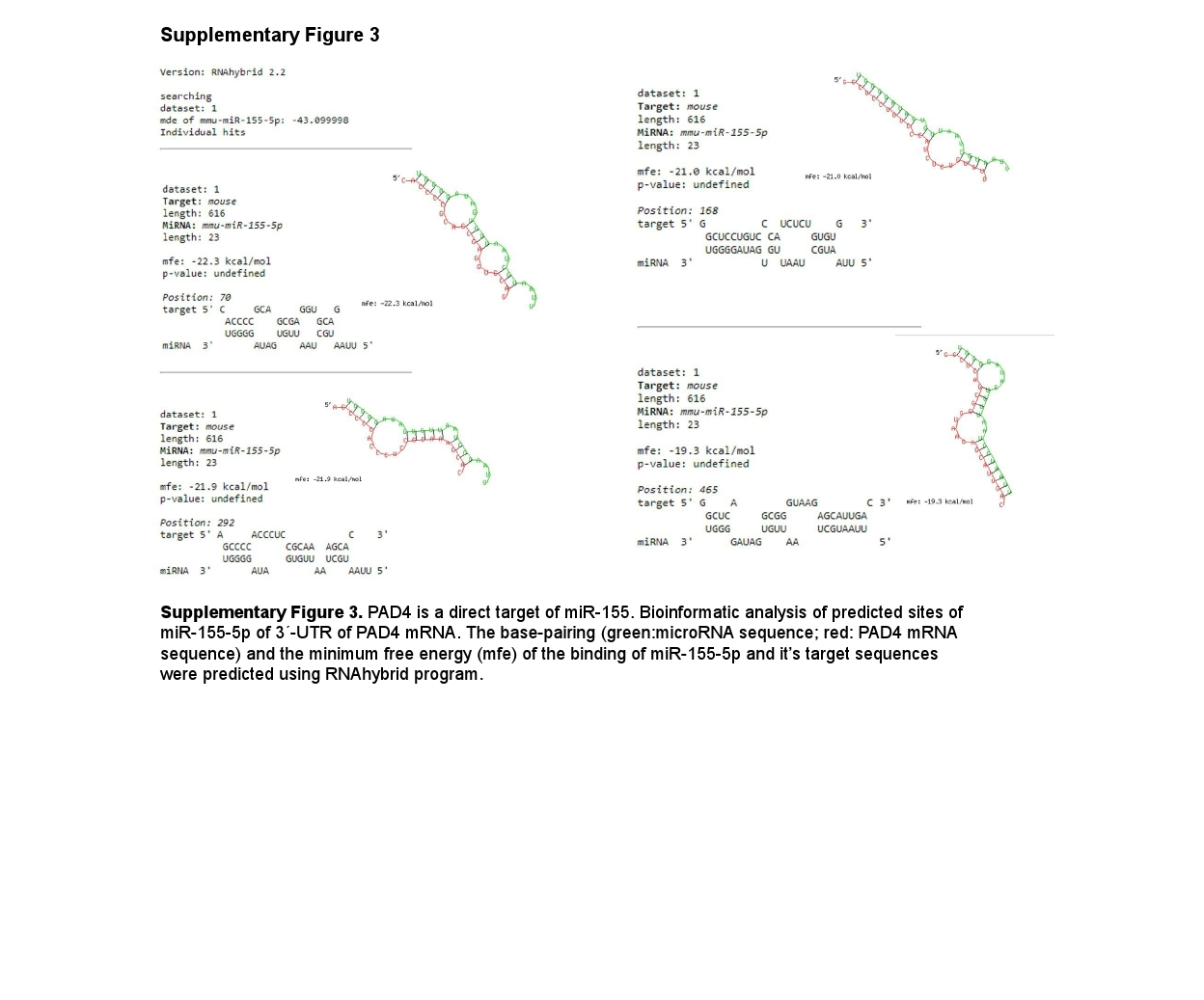

Supplement: Supplementary file 3 [file Image_3.tiff]

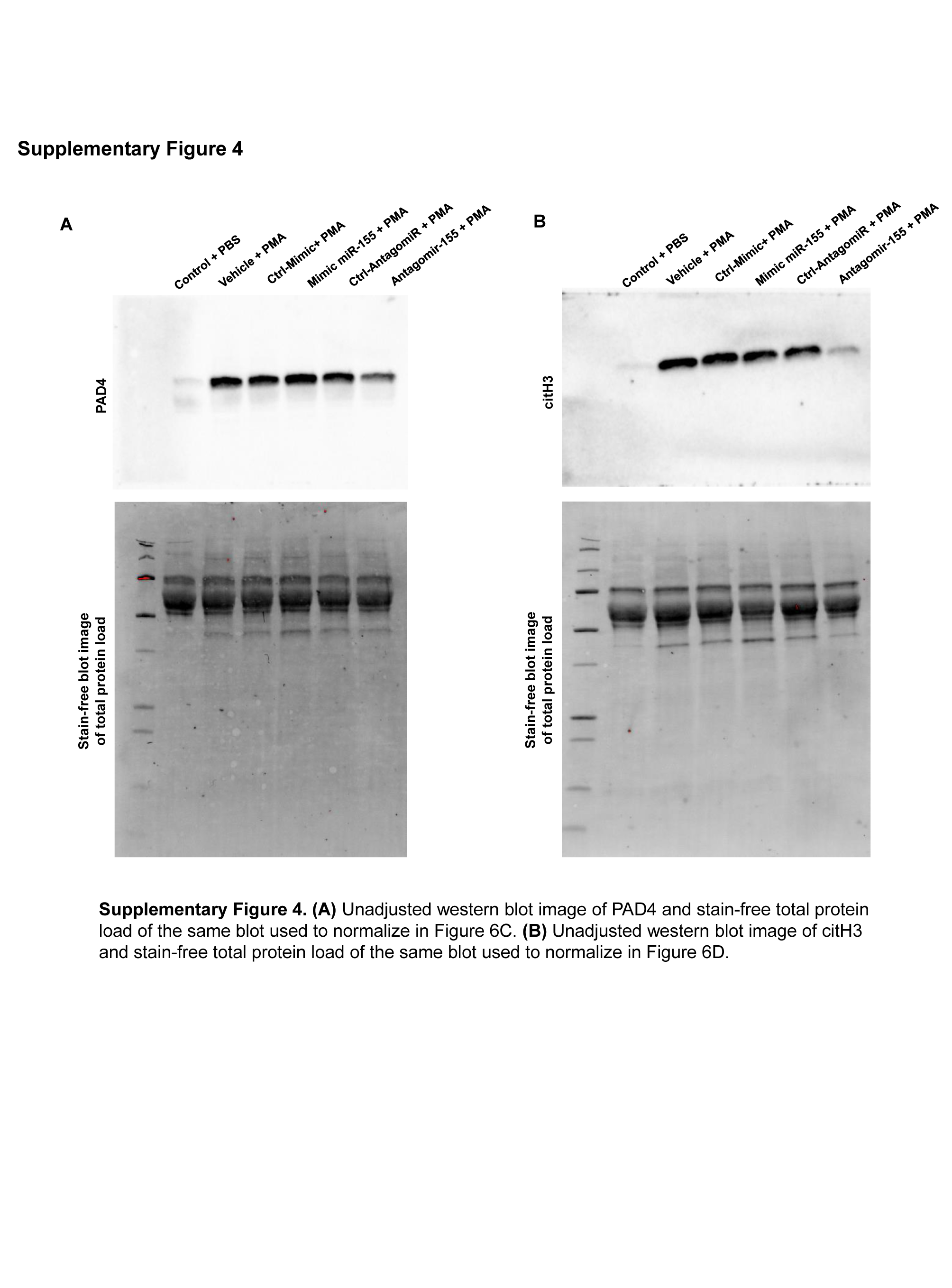

Supplement: Supplementary file 4 [file Image_4.tif]
